# Heritability and genome-wide associations studies of cerebral blood flow in the general population

**DOI:** 10.1177/0271678X17715861

**Published:** 2017-06-19

**Authors:** M Arfan Ikram, Hazel I Zonneveld, Gennady Roshchupkin, Albert V Smith, Oscar H Franco, Sigurdur Sigurdsson, Cornelia van Duijn, André G Uitterlinden, Lenore J Launer, Meike W Vernooij, Vilmundur Gudnason, Hieab HH Adams

**Affiliations:** 1 Department of Epidemiology, Erasmus MC, Rotterdam, the Netherlands; 2 Department of Radiology and Nuclear Medicine, Erasmus MC, Rotterdam, the Netherlands; 3 Department of Neurology, Erasmus MC, Rotterdam, the Netherlands; 4 Department of Medical Informatics, Erasmus MC, Rotterdam, the Netherlands; 5 Icelandic Heart Association, Kopavogur, Iceland; 6 Faculty of Medicine, University of Iceland, Reykjavik, Iceland; 7 Department of Internal Medicine, Erasmus MC, Rotterdam, the Netherlands; 8 Intramural Research Program, NIA, NIH, Bethesda, MD, USA

**Keywords:** Aging, cerebral blood flow, genome-wide association study, heritability, population-based

## Abstract

Cerebral blood flow is an important process for brain functioning and its dysregulation is implicated in multiple neurological disorders. While environmental risk factors have been identified, it remains unclear to what extent the flow is regulated by genetics. Here we performed heritability and genome-wide association analyses of cerebral blood flow in a population-based cohort study. We included 4472 persons free of cortical infarcts who underwent genotyping and phase-contrast magnetic resonance flow imaging (mean age 64.8 ± 10.8 years). The flow rate, cross-sectional area of the vessel, and flow velocity through the vessel were measured in the basilar artery and bilateral carotids. We found that the flow rate of the basilar artery is most heritable (h2 (SE) = 24.1 (9.8), *p*-value = 0.0056), and this increased over age. The association studies revealed two significant loci for the right carotid artery area (rs12546630, *p*-value = 2.0 × 10^−8^) and velocity (rs2971609, *p*-value = 1.4 × 10^−8^), with the latter showing a concordant effect in an independent sample (N = 1350, *p*-value = 0.057, meta-analyzed *p*-value = 2.5 × 10^−9^). These loci were also associated with other cerebral blood flow parameters below genome-wide significance, and rs2971609 lies in a known migraine locus. These findings establish that cerebral blood flow is under genetic control with potential relevance for neurological diseases.

## Introduction

The cerebral circulation consists of an intricate system of blood vessels that supply the brain with nutrients. The flow of blood to the brain parenchyma is tightly regulated,^1–3^ with deviations from this resulting in functional impairments. Both ischemia and hyperemia are associated with neurological diseases, including Alzheimer’s disease,^4^ other neurodegenerative disorders,^5^ stroke,^6^ and small vessel disease.^7^ It is therefore imperative to identify factors that influence cerebral blood flow.

Numerous environmental factors have been associated with cerebral blood flow, among which are physical activity,^8^ psychological stress,^9^ and high altitude.^10^ Some studies have also hinted at an influence of genetics, with reports of cerebral blood flow dysregulation in mutation carriers of diseases, such as the *COMT* Val158Met mutation^11^ and *HTT* CAG repeats.^12^ However, the extent to which genetics contributes to the variability in cerebral blood flow is yet to be determined. Furthermore, only small candidate gene studies have been published, while there have been no reports of unbiased genome-wide studies. Identification of genetic variants associated with cerebral blood flow could provide insight into neurological disorders that are related to flow abnormalities.

Here we systematically map the heritability of cerebral blood flow in a large, population-based cohort study, followed by genome-wide association studies and independent replication.

## Material and methods

### Study population

This study was embedded in the Rotterdam Study,^13^ a Dutch population-based cohort study including a total of 14,926 participants who were aged 45 years or over at enrolment. The cohort was designed to investigate causes and determinants of chronic diseases in the elderly, although participants were not selected for the presence of diseases or risk factors. The participants were recruited in three batches: in 1990 (RS-I), in 2000 (RS-II), and in 2006 (RS-III). Genotyping was successfully performed in 11,496 participants of European descent. Since 2005, all participants underwent brain magnetic resonance imaging (MRI) to examine the causes and consequences of age-related brain changes.^14^ Until 2013, a total of 5806 unique individuals were scanned, of which 4864 had genetic data available. The Rotterdam Study has been approved by the institutional review board of Erasmus MC University Medical Center (Medical Ethics Committee), in accordance with the Helsinki Declaration of 1975 (and revised in 1983), and in accordance with the Population Study Act Rotterdam Study, executed by the Ministry of Health, Welfare and Sports of the Netherlands. All participants provided written informed consent to participate in the study and to obtain information from their treating physicians.

### Genotyping

Genotyping was performed with the Illumina 550 K, 550 K duo, and 610 quad arrays.^13^ We then removed samples with call rate below 97.5%, gender mismatch, excess autosomal heterozygosity, duplicates or family relations and ancestry outliers, and variants with call rate below 95.0%, failing missingness test, Hardy–Weinberg equilibrium *p*-value <10^−6^, and minor allele frequency <1%. Genotypes were imputed to the 1000 Genomes phase I version 3 reference panel (all population) by using the MACH/minimac software.^15^

### Image acquisition and processing

MRI scanning was performed on a dedicated 1.5-T MRI unit with an eight-channel head coil (Signa HD platform, GE Healthcare, Milwaukee, USA). The MRI protocol included high-resolution axial sequences (T1-weighted, T2-weighted, and fluid attenuated inversion recovery (FLAIR)), as well as 2D phase-contrast imaging. A detailed description of the MRI protocol was presented elsewhere*.*^14^ All scans were visually inspected by trained raters for cortical infarcts and, if present, the scan was excluded since infarcts may have a large effect on the measurements of cerebral blood flow (N = 196).

For cerebral blood flow measurement, 2D phase-contrast imaging was performed as described previously.^16^ In short, a sagittal 2D phase-contrast MRI angiographic scout image was performed. On this scout image, a transverse imaging plane perpendicular to both the precavernous portion of the internal carotid arteries and the middle part of the basilar artery was chosen for a 2D gradient-echo phase-contrast sequence (repetition time = 20 ms, echo time = 4 ms, field of view = 19 × 19 cm^2^, matrix = 256 × 160, flip angle = 8°, NEX = 8, bandwidth = 22.73 kHz, velocity encoding = 120 cm/s, slice thickness = 5 mm). Acquisition time was 51 s and no cardiac gating was performed.^17^ Measures of cerebral blood flow were derived from the phase-contrast images by using interactive data language-based custom software (Cinetool version 4; General Electric Healthcare). Two experienced MRI technicians manually marked the basilar artery as well as the left and right carotid arteries with excellent agreement (inter- and intra-rater correlation both previously determined to be >0.94).^16^ The cross-sectional area of these vessels (cm^2^) and flow velocity (cm/sec) through them were determined from these regions of interest. The flow rate (mL/s) for each vessel was calculated from the area and velocity. Additionally, the total cerebral blood flow was calculated by summing flow rates for the basilar and carotid arteries and expressed in mL/min. Details on the assessment of cerebral blood flow can be found in a previous report.^16^ Outlying values of more than 3.5 standard deviation from the mean were excluded from the analysis to prevent effects being driven by a few individuals. Finally, we performed a rank normal transformation of the data to ensure that the variables are in a normal distribution, since the heritability estimation is sensitive to deviations from this.

To determine brain volume, T1 images were segmented into supratentorial gray matter, white matter and cerebrospinal fluid by using a k-nearest neighbor algorithm.^18^ White matter lesions were segmented by using the T1 tissue maps and an automatically detected threshold for the intensity of FLAIR scans.^19^ Visual inspection of the segmentations led to the exclusion of 197 poor quality scans, leaving 4472 individuals for analysis.

### Heritability analysis

To estimate heritability in our sample of unrelated individuals, we implemented Genome-wide Complex Trait Analysis (GCTA).^20^ Briefly, this method compares genetic similarity between individuals with phenotypic similarity to estimate the amount of variance explained by genetics. For this, a genetic relationship matrix was calculated as previously described;^21^ 1000 Genomes imputed genotypes were filtered on imputation quality (R^2 ^< 0.5) and allele frequency (MAF < 0.01) and used to calculate pairwise genetic relatedness between all individuals. For pairs with more than 0.02 genotype similarity, one individual was randomly removed.

We estimated the heritability of the area, velocity, and flow for each of the three vessels, as well as the total cerebral blood flow. All analyses were adjusted for age, age^2^, sex, intracranial volume (model 1) and, to take into account any atrophy, additionally for the total supratentorial brain tissue volume (model 2). To determine patterns of heritability across the age range of our population, we performed a sliding window analysis. Individuals were ranked on the basis of their age and the heritability analyses were performed in the youngest 2000. Next, the study population shifted in 10 equal steps to the oldest 2000 individuals.

### Genome-wide association analysis

Genome-wide association analyses were performed on the cerebral blood flow parameters for the first model by using the HASE software.^22^ Variants were removed if they had an R^2 ^< 0.5 and an MAF < 0.05. Analyses were performed in the three subcohorts of the Rotterdam Study separately and meta-analyzed with the METAL software.^23^ Variants with a *p*-value <5 × 10^-8^ were considered genome-wide significant.

### Replication

Replication of genome-wide significant variants was performed in the Age, Gene/Environment Susceptibility (AGES) study, a population-based study in Reykjavik, Iceland, and which is described in detail elsewhere.^24^ Briefly, genotyping was performed with the Illumina HumanCNV370 chip and data were imputed to the same 1000 Genomes reference panel. Cerebral blood flow was measured in a similar way by using 2D gradient-echo phase contrast sequence (FOV 220 mm, matrix 25 × 256, TE 6.2 ms, TR 20 ms, flip angle 9°, velocity encoding 100 cm/s, slice thickness 5 mm). The images were analyzed using the software package FLOW^25^ by a single investigator (S.S.), with excellent agreement between repeated cerebral blood flow measurements in 20 scans (coefficients of variation 1.7).

## Results

### Study population

The characteristics of the population included in this study are shown in Table 1. The mean age was 64.8 (standard deviation 10.8) years, ranging from 45.7 to 97.9. The total brain volume was on average 82% of intracranial volume. The majority of the cerebral blood flow was provided by the carotids (80%), which was comparable between left and right.

**Table 1.** Study population characteristics.

Characteristic	Rotterdam Study (N = 4472)	AGES- Reykjavik (N = 1350)
Age, in years	64.8 ± 10.8	79.7 ± 4.7
Men, N (%)	1981 (44.3%)	513 (38.0%)
Intracranial volume, in cm^3^	1141.4 ± 114.9	1485.8 ± 144.0
Brain volume, in cm^3^	937.5 ± 100.9	1060.3 ± 97.1
Flow rate, in cm^3^/s		
Basilar artery	1.7 ± 0.6	1.0 ± 0.74
Left carotid	3.5 ± 0.9	2.9 ± 1.0
Right carotid	3.5 ± 0.9	3.0 ± 1.0
Vessel cross-sectional area, in cm^2^		
Basilar artery	0.29 ± 0.05	0.24 ± 0.06
Left carotid	0.35 ± 0.07	0.35 ± 0.07
Right carotid	0.36 ± 0.07	0.36 ± 0.08
Flow velocity, in cm/s		
Basilar artery	11.2 ± 4.3	7.5 ± 2.9
Left carotid	15.2 ± 4.6	10.2 ± 2.8
Right carotid	14.9 ± 4.5	10.4 ± 3.1

Note: Values represent mean ± standard deviation, unless otherwise stated.

### Heritability analysis

Table 2 shows the heritability estimates of cerebral blood flow. The flow through the vessel, rather than the area or the velocity, was the most heritable parameter. The highest heritability was for the basilar artery (h2 = 21.8) followed by the total cerebral blood flow (h2 = 16.1). Additional adjustment for brain volume slightly increased the heritability estimates.

**Table 2.** Heritability of cerebral blood flow parameters in the Rotterdam Study.

		Model 1		Model 2	
Parameter	N	h2 (SE)	*P*	h2 (SE)	*P*
Total cerebral blood flow	3485	16.1 (9.8)	0.048	17.8 (9.8)	0.033
Flow rate					
Basilar artery	3489	21.8 (9.8)	0.011	24.1 (9.8)	0.0056
Left carotid	3480	9.5 (9.8)	0.17	10.4 (9.8)	0.15
Right carotid	3481	13.8 (9.9)	0.081	9.9 (9.8)	0.16
Vessel area					
Basilar artery	3475	9.0 (9.2)	0.14	9.7 (9.2)	0.13
Left carotid	3482	0.0 (9.4)	0.50	0.0 (9.4)	0.50
Right carotid	3482	0.0 (9.6)	0.50	0.0 (9.6)	0.50
Flow velocity					
Basilar artery	3451	12.1 (9.8)	0.10	13.4 (9.8)	0.082
Left carotid	3445	7.5 (9.9)	0.23	8.5 (10.0)	0.20
Right carotid	3449	3.5 (9.6)	0.36	3.5 (9.6)	0.36

Note: Heritability estimates of cerebral blood flow parameters under two models: adjusting for age, age^2^, sex, intracranial volume (model 1) and additional for brain volume (model 2).

h2 = heritability; N = sample size; SE = standard error*.*

**Figure 1.**
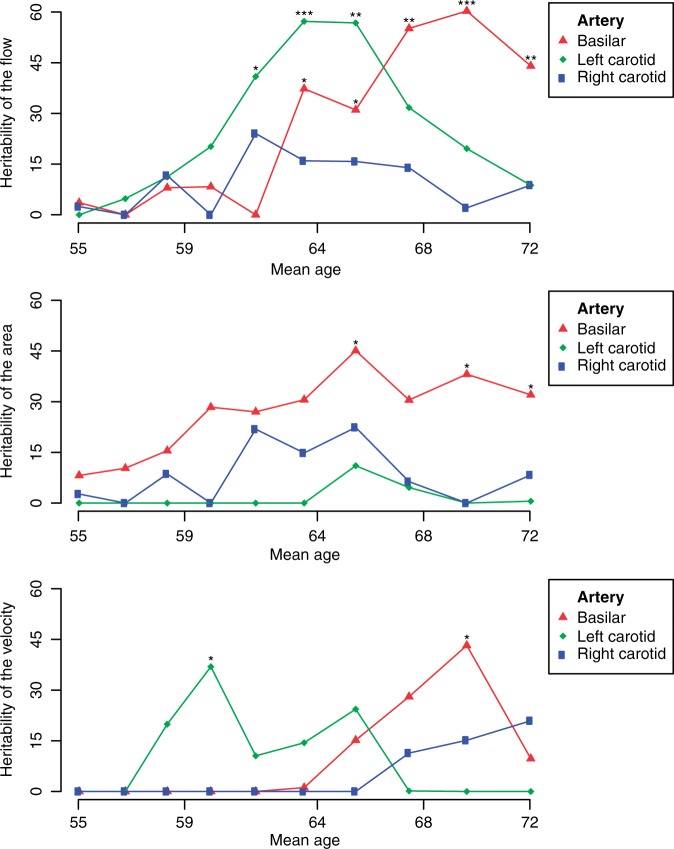
Heritability of cerebral blood flow parameters across age. A sliding window approach showing the heritability of the flow rate (top), vessel area (middle), and flow velocity (bottom) for three major cerebral arteries: the basilar artery in red, the left carotid artery in green, and the right carotid artery in blue. The results are adjusted for age, age^2^, sex, and intracranial volume (model 1). The total population consisted of 4472 individuals and the sliding window had a size of 2000 individuals (around 1750 after removing related individuals, see Supplementary Table S1). We passed through the total population in 10 steps, i.e. moving by an average of 274 individuals in each step.

By using the sliding window approach, we found that the heritability was higher in the older age groups, most noticeable for the basilar artery parameters (Figure 1, Supplementary Table S1). The results were similar after adjustment for total brain volume (Supplementary Figure 1).

### Genome-wide association analysis

**Figure 2.**
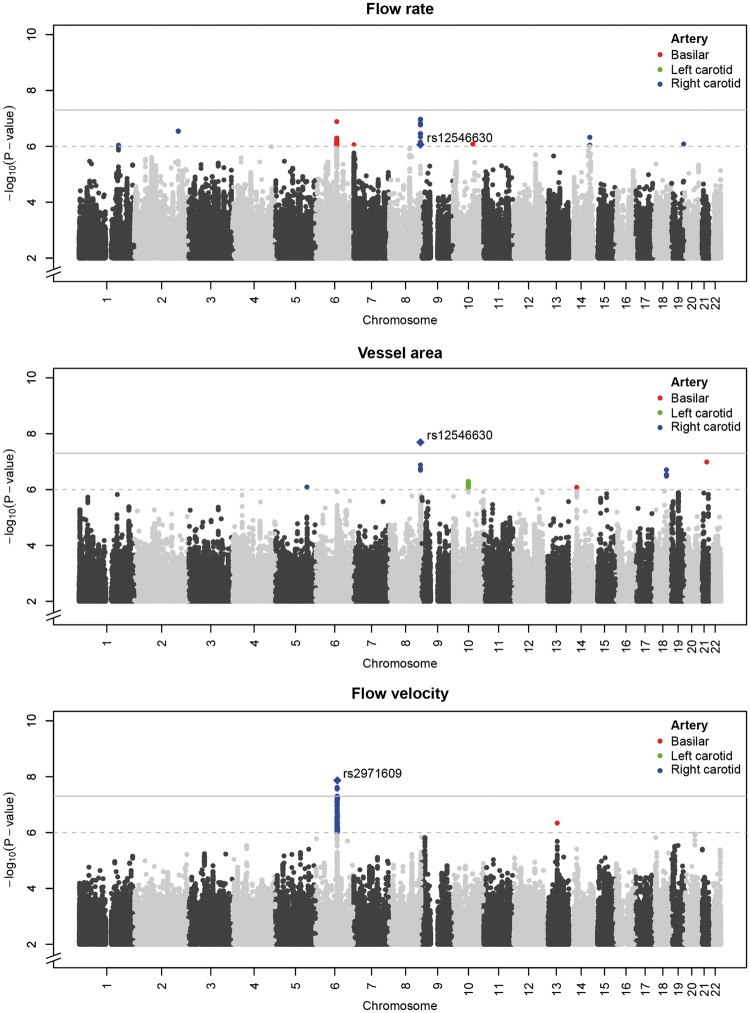
Genome-wide association studies of cerebral blood flow. Manhattan plots for the genome-wide association studies of the flow rate (top), vessel area (middle), and flow velocity (bottom) for three major cerebral arteries: variants with *p-*values <10^−6^ are indicated in red for the basilar artery, green for the left carotid, and blue for the right carotid.

**Figure 3.**
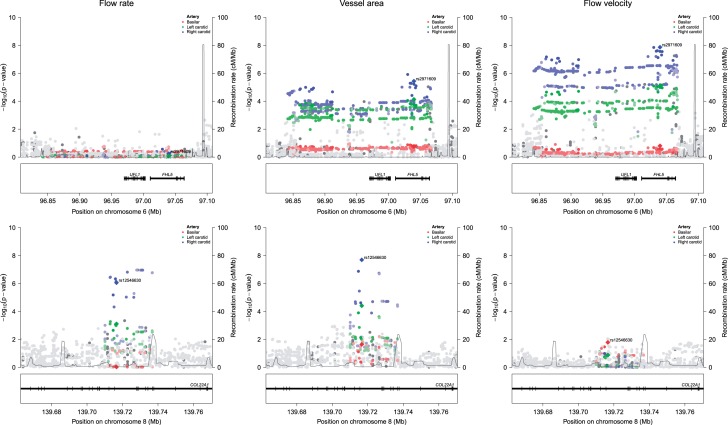
Top genetic loci for cerebral blood flow. Regional association plots of the two genome-wide significant loci, 6q16.1 (upper row) and 8q24.23 (lower row). Associations are shown with the flow rate (left), vessel area (middle), and flow velocity (right) for three major cerebral arteries: the basilar artery in red, the left carotid artery in green, and the right carotid artery in blue.

Next, we performed genome-wide association studies of cerebral blood flow parameters and identified two significant loci (Figure 2, all variants with *p*-values <10^−6^ are provided in Supplementary Table S2). These were 6q16.1 for the flow velocity through the right carotid (rs2971609, *p*-value = 1.4 × 10^−8^) and 8q24.23 for the cross-sectional area of the right carotid (rs12546630, *p*-value = 2.0 × 10^×8^). Both top variants were intronic (Figure 3), with 6q16.1 being a known locus for migraine.^26^ When examining the two loci in relation to all cerebral blood flow parameters (Table 3), we found that the 6q16.1 locus was associated with a larger area and a lower velocity in both carotids, while 8q24.23 was associated with larger area and flow rate of both carotids. The results did not differ materially after further adjustment for brain volume (Supplementary Table S3).

**Table 3.** Association of genome-wide significant loci with cerebral blood flow parameters in the Rotterdam Study.

		rs12546630		rs2971609	
Parameter	N	β (SE)	*P*	β (SE)	*P*
Total cerebral blood flow	4453	.098 (.022)	7.41 × 10^−6^	.005 (.022)	0.8141
Flow rate					
Basilar artery	4462	−.002 (.025)	0.9429	−.005 (.024)	0.849
Left carotid	4453	.080 (.024)	7.74 × 10^−4^	.004 (.024)	0.8661
Right carotid	4453	.116 (.024)	8.71 × 10^−7^	.013 (.024)	0.5807
Vessel area					
Basilar artery	4440	.056 (.025)	0.02272	.034 (.025)	0.1683
Left carotid	4450	.100 (.024)	3.9 × 10^−5^	.096 (.024)	7.32 × 10^−5^
Right carotid	4454	.137 (.024)	2.02 × 10 ^−8^	.112 (.024)	3.67 × 10^−6^
Flow velocity					
Basilar artery	4420	−.057 (.024)	0.01628	−.034 (.024)	0.1554
Left carotid	4415	−.033 (.022)	0.136	−.097 (.022)	7.88 × 10^−6^
Right carotid	4418	−.033 (.022)	0.1302	−.123 (.022)	1.36 × 10^−8^

Note: Effect estimates of the two genome-wide significant loci with cerebral blood flow parameters, adjusting for age, age^2^, sex, and intracranial volume (model 1).

h2: heritability; N: sample size; SE: standard error.

### Replication

We attempted replication of the two significant loci in an independent cohort and found a borderline significant and concordant signal for 6q16.1 for the flow velocity through the right carotid (rs2971609, *p*-value = 0.057), which is increasing in significance after meta-analysis (*p*-value = 2.5 × 10^−9^). There was no apparent association for 8q24.23 in the replication cohort (*p*-value = 0.83, beta in opposite direction).

## Discussion

We find that, in the general population, the total cerebral blood flow is partly determined by genetic factors, with the highest heritability for the flow through the basilar artery. The genetic contribution tends to be more prominent at older age (>65 years). We also identify the first genome-wide significant loci for cerebral blood flow, one of which showing a similar effect in an independent cohort.

While we are not aware of other heritability studies of cerebral blood flow, the fact that it is heritable should not be surprising given many reports on the heritability of brain structure. Most such studies were performed in twins, which produce systematically higher estimates than the population-based approach we used here.^20^ Indeed, most heritability estimates of brain traits were considerably higher. In contrast, the few studies in unrelated individuals that used common genotyped variants reported substantially lower heritabilities than the twin-based approach.^27–30^ Nevertheless, the proportion of the phenotypic variance explained by genetics is less than half that of height, which is estimated at 60% following a similar approach to our study.^31^ Cerebral blood flow might therefore be more susceptible to environmental factors such a person’s lifestyle, which could in turn influence cardiovascular health. If some of these consequences are vessel-specific, this may help to explain the differences between basilar artery and the carotids.

Besides the flow rate, we also examined its two components: the vessel’s cross-sectional area and the flow velocity. Interestingly, these were both less heritable than the flow rate. One explanation for this is that flow needs to be constant to provide the brain with sufficient nutrients, while the area or velocity may vary as each can compensate the other. Since flow is most crucial for the brain to function adequately, this might explain why it is under tighter genetic control. Alternatively, there could be more intra-individual variation in the area and velocity compared to flow, also resulting in heritability differences between the parameters.

Another intriguing finding was that flow parameters were generally more heritable with increasing age. This trend was most apparent for the basilar artery, reaching heritability estimates over 60% around 70 years of age. Environmental exposures accumulate over the lifetime while a person’s genetic make-up remains stable. It is therefore generally thought that the relative contribution of genetics to the phenotypic variance might decrease over time, but this is not well established in humans. In a multi-generational cohort, four complex traits were studied but there was little evidence for differences over time in their heritability.^32^ For cognitive ability, a gradual increase in the heritability has been reported from infancy to adulthood,^33–36^ and it remains high even in persons over 80 years old.^37^ The observed increase in the heritability of cerebral blood flow over age could have several explanations. It is possible that the genetic contribution remains stable but that environmental factors become less important with increasing age. For example, the amount and variation in physical exercise might decrease in the elderly, thereby causing an apparent increase in the heritability. Also, some genetic factors might exert their effect on cerebral blood flow (more strongly) later in life, with a notable example of such a gene being *APOE*. Additionally, pathology-driven changes in the cerebral vasculature and parenchyma, e.g. due to Alzheimer’s disease or stroke, might make the cerebral blood flow more dependent on compensatory mechanisms. Their effects might express only late in life and in turn the heritability of the response reaction cannot emerge early in life.

Heritability, which measures the effect of all genetic variants, does not necessarily imply greater success in gene discovery, where each genetic variant is studied separately. This is illustrated by the fact that our genome-wide significant findings were for the area and velocity of the right carotid artery, parameters which were not significantly heritable. The top variants for the two significant loci were located in the introns of *FHL5* (6q16.1) and *COL22A1* (8q24.23). *FHL5*, four-and-a-half LIM domains 5, encodes a transcription factor and was previously identified for migraine,^26^ and subsequently associated with cervical artery dissection.^38^ The gene is important for the proliferation of vascular smooth muscle cells,^39^ pointing to a vascular mechanism through which it could increase susceptibility to these diseases. Furthermore, a meta-analysis of 375,000 individuals recently revealed that migraine loci are enriched for genes expressed in vascular and smooth muscle tissues, largely in line with its proposed vascular etiology.^40^ *COL22A1*, collagen type XXII alpha 1, is part of the collagen protein family and variants in this gene have been associated with serum creatinine^41^ and bronchodilator response in asthma,^42^ although these specific variants did not associate with cerebral blood flow in our study. While little is known about the function of *COL22A1*, currently unpublished results suggest it is important for maintaining vascular integrity and mutations cause intracranial aneurysms.^43^

The strengths of our study include its population-based setting and comprehensive investigation of cerebral blood flow parameters beyond the conventionally studied flow rate. Nevertheless, there are several limitations. First, the sample is relatively small. This is reflected in the standard errors of the heritability estimates, especially for the sliding window analysis, and this should caution against overinterpretation of the results. For the association analyses, we countered the small sample size by performing a replication study in an independent cohort. While we are unware of other population-based studies that have measured cerebral blood flow and genetics, larger collaborative studies should be undertaken once these data become available. Second, our approach for estimating the heritability in a sample of unrelated individuals by using genotyped variants represents the narrow sense heritability.^20^ This means that non-additive effects and the effects of untagged causal variants will be disregarded, thereby resulting in a potential underestimate. On the other hand, simple family-based models have recently been shown to inflate heritability estimates by almost 50% on average compared to estimates obtained from structural equation modelling.^44^ Given the methodological considerations for determining which part of the phenotypic variance is due to genetics, our findings can provide a lower bound of the true heritability of cerebral blood flow. Third, when additionally adjusting our analyses for total brain volume, we only used the volume of the supratentorial grey and white matter. This means that the cerebellum and brainstem, areas that receive blood from the basilar artery, were unaccounted for. We do not believe that this significantly influenced our findings since there were only marginal differences (which generally were improvements) when incorporating brain volume into the models. Fourth, we used phase-contrast imaging to measure cerebral blood flow, but more sophisticated methods are available, which also generate regional flow measures. Functional MRI, arterial spin labeling, and positron emission tomography are techniques that provide more detailed information on regional blood flow and perfusion. Furthermore, longitudinal measurements, both across the cardiac cycle (e.g. the peak systolic velocity) and across the lifespan, could be valuable in understanding the role of genetics in differences over time in cerebral blood flow. Finally, our study was included in persons of European descent, making the results not readily generalizable to other ethnicities.

In conclusion, our study establishes cerebral blood flow as a trait with a complex genetic basis. Larger studies can elucidate the genes underlying this intricate process in the brain and perhaps further our understanding of related neurological diseases.

## Supplementary Material

Supplementary Figure

Supplementary material

Supplementary Table
