# Supplementary figures and images for: Heritability and genome-wide associations studies of cerebral blood flow in the general population
Source: J Cereb Blood Flow Metab. 2017 Jun 19;38(9):1598–608. doi: 10.1177/0271678X17715861 (PMC6120124; doi:10.1177/0271678X17715861)

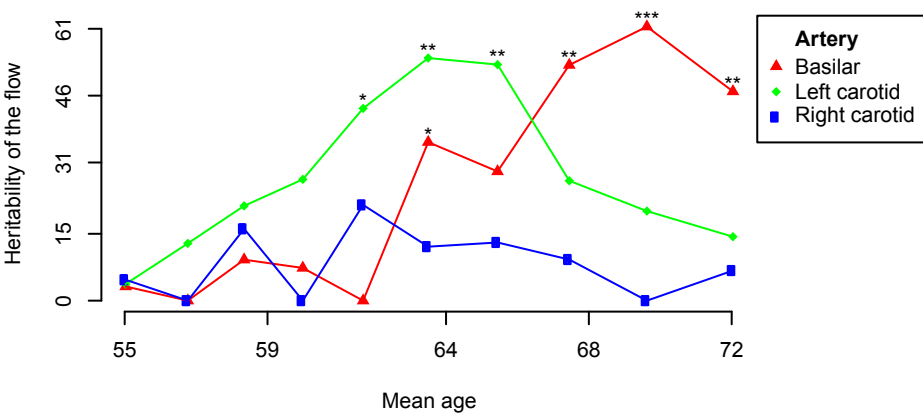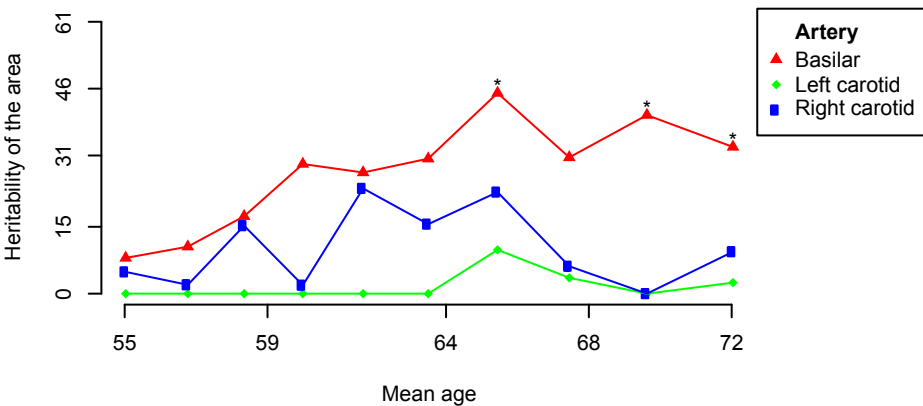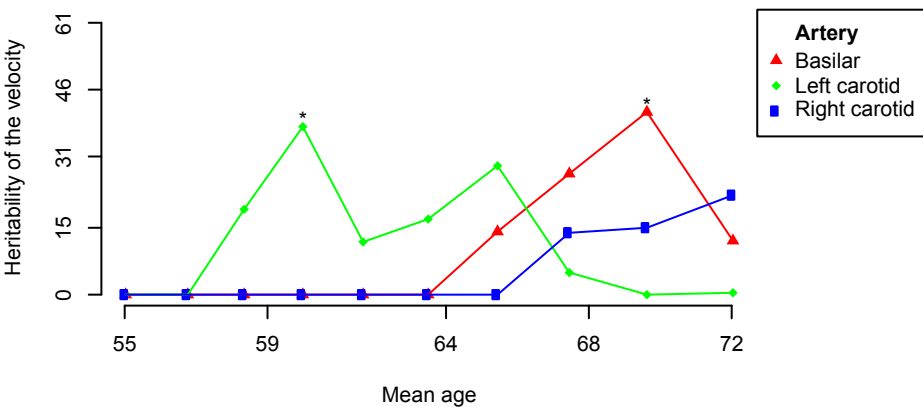

Supplement: Supplementary Figure [file JCB715861_supplementary_figure.pdf]
