# Supplementary material for: Heritability and genome-wide associations studies of cerebral blood flow in the general population
Source: J Cereb Blood Flow Metab. 2017 Jun 19;38(9):1598–608. doi: 10.1177/0271678X17715861 (PMC6120124; doi:10.1177/0271678X17715861)
Supplement: Supplementary material [file JCB715861_supplementary_material.pdf]

SUPPLEMENTARY FIGURE LEGEND

Supplementary Figure S1 | Heritability of cerebral blood flow parameters across age.

A sliding window approach showing the heritability of the flow rate (top), vessel area (middle), and flow velocity (bottom) for three major cerebral arteries: the basilar artery in red, the left carotid artery in green, and the right carotid artery in blue. The results are adjusted for age, age^2, sex, intracranial volume, and total brain volume (model 2). The total population consisted of 4472 individuals and the sliding window had a size of 2000 individuals (around 1750 after removing related individuals, see Supplementary Table S1). We passed through the total population in 10 steps, i.e. moving by an average of 274 individuals in each step.
